# Longitudinal relationship between screen time, cardiorespiratory fitness, and waist circumference of children and adolescents: a 3-year cohort study

**DOI:** 10.1186/s12887-023-04378-3

**Published:** 2023-11-04

**Authors:** Aline Rosso Lehnhard, Ana Paula Sehn, João Francisco de Castro Silveira, Ryan Donald Burns, Cézane Priscila Reuter, Silvia Isabel Rech Franke

**Affiliations:** 1 Graduate Program in Health Promotion, University of Santa Cruz do Sul, Santa Cruz do Sul, RS Brazil; 2 Graduate Program in Human Movement Sciences, Federal University of Rio Grande do Sul, Farroupilha, RS Brazil; 3 Department of Exercise and Sport Science, University of Utah, Salt Lake City, USA; 4 Health Promotion, University of Santa Cruz do Sul, Independência Av, 2293 – Universitário, Santa Cruz do Sul, RS 96815-900 Brazil

**Keywords:** Physical fitness, Sedentary behavior, Obesity, Youth, Cardio metabolic health

## Abstract

**Purpose:**

The aim of this study was to examine the interaction between screen time and cardiorespiratory fitness (CRF) in their longitudinal association with waist circumference (WC) during a follow-up of 3 years from childhood to adolescence.

**Methods:**

Observational 3-year longitudinal study with 401 students (224 females), seven to 15 years of age at baseline. The CRF was evaluated by estimating peak oxygen uptake (VO_2peak_) from an indirect field-based test and body mass index. Screen time was assessed using self-reported questionnaires. Moderation analyses were tested using a multiple linear regression model with adjustments for sex, age, puberty stage, and ethnicity.

**Results:**

A statistically significant interaction term was observed (B = -0.0003; 95% CI: -0.007; -0.0001). Since screen time was contextualized as the independent variable, the results show that relationship between screen time and WC varies across different CRF levels.

**Conclusion:**

The findings suggest that higher CRF can attenuate the harmful association that increased sedentary behavior might have on abdominal adiposity.

## Introduction

Overweight in childhood has well-known harmful effects on general health, such as biological, physical, mental and social alterations [1]. Considering the detrimental health effects associated with body adiposity, it becomes crucial to monitor adiposity levels in children and adolescents. Therefore, we emphasize the importance of assessing waist circumference (WC), which is a widely utilized measure of adiposity that effectively indicates the accumulation of abdominal fat and consequently serves as a reliable predictor of body fat distribution among children and adolescents. Additionally, WC has demonstrated to be associated with cardiometabolic diseases, including insulin resistance, dyslipidemia, and liver disease [2, 3].

The alteration in the weight status of schoolchildren is influenced by various factors, among which are lifestyle habits such as sedentary behavior, as well as levels of physical fitness [4]. It is accepted that sedentary behavior, particularly characterized by extensive screen usage, is detrimental to optimal health. This is notably linked to unfavorable body composition, excess body weight, obesity, and metabolic syndrome [5], and it represents a harmful health behavior [6]. From this perspective, it is encouraged for children and adolescents to adhere to sedentary behavior guidelines, which typically advise them to limit their recreational screen time [5].

On the other hand, physical fitness is also emphasized, especially cardiorespiratory fitness (CRF), as it stands out as one of the most important health markers [7]. It is well accepted that maintaining optimal CRF levels are associated with several health benefits during childhood and adolescence. These advantages encompass a reduced risk of cardiovascular disease, certain types of cancer, premature mortality, and hypertension [8]. It is well-established that higher levels of CRF are associated with reduced adiposity, circulating glucose, and lipid levels [9]. Furthermore, CRF may also influence pulmonary function, mental well-being, and bone health [8]. However, recent decades have witnessed a decline in CRF levels worldwide. With respect to Brazilian children and adolescents, a systematic review [10] has described that among a total of 49,093 individual aged six to 19 years, only 32.2% of them exhibit healthy CRF levels.

Lower CRF levels in children and adolescents, independently from screen time, were identified as a risk factor for unfavorable measures of body fat and abdominal adiposity. Conversely, an excess time in front of the screens was shown to be associated with higher WC, independently from CRF levels of students [11]. Moreover, it has been observed that adolescents with metabolic syndrome exhibit longer recreational screen time and lower CRF [12]. These findings reinforce the negative impact of high exposure to screens and low CRF, on both the current and long-term health of children and adolescents.

Despite the existing literature linking CRF and screen time with WC, the interaction between these two exposures has not been thoroughly investigated. Previous cross-sectional data have demonstrated how CRF and screen time interact in their association with adiposity parameters [13]. However, the evolution of this moderation effect from childhood to adolescence remains insufficiently understood. There is an urgent need to understand how the variables evolve and interact over the years in order to implement more effective interventions aimed to improve CRF, reduce screen time, and decrease WC. Thus, the present study aimed to examine the interaction between screen time and CRF in their longitudinal association with WC during a follow-up of 3 years from childhood to adolescence.

## Method

### Participants

This was an observational 3-year longitudinal study with 401 Brazilian students, seven to 15 years of age at baseline. The sampling process has been described elsewhere [14]. Briefly, all children from 25 randomly selected schools were invited to participate at the beginning of the study (n = 20,380). The initial sample consisted of those whose parents or guardians gave permission in writing for them to participate (n = 1,687), representing a recruitment success of 8.3%. All participants were contacted for follow-up after 3 years, but only 401 participants had completed data for the present analysis (76% of loss from baseline to follow-up). The present study was approved by the Ethics Committee on Research with Human Beings of the University of Santa Cruz do Sul (UNISC), under protocol no. 3644.667 and CAAE: 23199619.5.0000.5343.

The sample size of the present study was calculated using the G*Power software version 3.1.9.7. A small to medium effect size (f^2^ = 0.07) was used in addition to an α probability of 0.05, 80% of power, and six predictors. A total sample size of 202 participants was established for the regression model.

### Procedures

Trained professionals from the University of Santa Cruz do Sul performed all evaluations.

#### Ethnicity

The ethnicity was self-reported by the participants.

#### Cardiorespiratory fitness

The CRF measurement was evaluated using an estimation of peak oxygen uptake (VO_2peak_) from indirect running and walking field-tests and body mass index (BMI) assessment. The tests used at baseline and at follow-up were performed according to the protocols of PROESP-Br (2009) [15] and PROESP-Br (2012) [16]. Specifically, the duration of the tests were 9 and 6 min at baseline and follow-up, respectively. The estimation of VO_2peak_ at baseline was calculated using the following equation: VO_2peak_ = 47.547 + 0.008(distance in meters) − 0.805(BMI) + 4.236(sex) [17]; whereas, at follow-up the following equation was used: VO_2peak_ = 41.946 + 0.022(distance in meters) − 0.875(BMI) + 2.107(sex) [18]. Both equations used 0 for females and 1 for males.

#### Screen time

The assessment of the recreational time spent in front of screens was performed by a self-reported questionnaire. In this instrument, participants were required to record the amount of daily time (minutes) they dedicated to activities such as watching television, playing video games, and using the computer. The reported times for each activity were then summed to determine the overall screen time.

#### Waist circumference

WC measurements process involved identifying the narrowest part of the trunk between the ribs and the iliac crest and measure the perimeter from that area using an inelastic measuring tape with a precision of 1 mm (Cardiomed®). The cutoff points of Fernández et al. [19], which consider WC to be at risk when the waist perimeter is above the 75th percentile taking biological sex and age into account, were used to describe WC levels.

#### Puberty stage

The time before or after the peak height velocity (PHV), measured in years, was estimated using the equations proposed by Moore et al. [20] for males: PHV = -7.999994 + 0.0036124 * age * height; and for females: -7.709133 + 0.0042232 * age * height.

### Statistical analyses

Statistical analysis was performed using the Statistical Package for the Social Sciences (SPPS) version 23.0 software (IBM, Armonk, NY). Descriptive statistics at baseline and at follow-up were expressed using means and confidence intervals (CI) for continuous variables; whereas the categorical variables were expressed using absolute and relative frequencies. The bootstrapping resampling procedure with the bias-corrected and accelerated (BCa) method using 5,000 samples for the paired-sample *t*-test was used to verify differences between the two time-points. Standardized differences, measured by the delta (d) of Cohen, were calculated to identify the magnitude of difference. Values of *d* < 0.49 indicate a small difference; 0.50 ≤ *d* ≤ 0.79 indicated a medium difference; and *d* ≥ 0.80 indicated a large difference [21].

The PROCESS (version 4.2) macro for SPSS was used to test moderation analyses [22] using a multiple linear regression model. The model of PROCESS number 1 was used to estimate WC values from different predictors: screen time, CRF (VO_2peak_), interaction between screen time and CRF, PHV, ethnicity, and measurement period. Before analysis, WC and VO_2peak_ were converted into z-scores using international sex- and age-specific reference values [23]. Therefore, there was no need to adjust the multiple linear regression model for sex and age. Also, a variable named ‘measurement period’ was computed to account for the amount of time between the two time-points of assessment. The dataset was restructured to a long format to include the variables’ observations from both assessment moments in the analysis. The Johnson-Neyman technique was used to better understand the relationship between screen time across different CRF values. All inferences were made based on the 95% CI.

## Results

The descriptive characteristics of the sample are presented in Table 1. After 3-years of follow-up, there was an increase in the mean values of BMI, screen time, VO_2peak_, WC, and PHV. Among these variables, only VO_2peak_ values demonstrated a small increase (*d* = 0.24), while the remaining variables exhibited a large increase (*d* ≥ 1.00).

**Table 1:** Descriptive characteristic of the sample

	**2011**	**2014**	**Standardized difference**
	**Baseline**	**3-y Follow-up**	
	**Mean (95% CI)**	**Mean (95% CI)**	**Cohen’s** ***d***
Age (years)	10.47 (10.27; 10.67)	13.14 (12.97; 13.33) *	7.00
Weight (kg)	39.78 (38.51; 41.04)	52.45 (51.05; 53,90) *	1.99
Height (m)	1.43 (1.42; 1.44)	1.57 (1.56; 1.58) *	10.82
BMI (kg/m^2^)	19.98 (18.60; 19.35)	21.02 (20.58; 21.43) *	1.00
Screen time (min/day)	223.99 (208.47; 240.36)	232.87 (214.33; 251.22)	0.04
VO_2peak_ (mL.min^− 1^.kg^− 1^)	44.16 (43.71; 44.59)	45.10 (44.47; 45.80) *	0.24
WC (cm)	64.00 (63.06; 65.00)	69.65 (68.59; 70.76) *	1.04
PHV (years)	-1.82 (-2.00; -1.64)	0.38 (0.20; 0.57) *	5.13
***n*** **(%)**			
*BMI*			
Underweight	5 (1.2)	4 (1.0)	
Normal Weight	239 (59.6)	243 (60.6)	
Overweight	76 (19.0)	74 (18.5)	
Obesity	81 (20.2)	80 (19.1)	
*WC Classification*			
Low risk	297 (74.1)	307 (76.6)	
High risk	104 (25.9)	94 (23.4)	
*Screen time*			
≤ 2 h/day	150 (37.4)	148 (36.9)	
>2 h/day	251 (62.6)	253 (63.1)	
*Sex*			
Male	177 (44.1)		
Female	224 (55.9)		
*Ethnicity*			
White	316 (78.8)		
Non-white	85 (21.2)		

Notes – CI: confidence interval; n: absolute frequency; %: relative frequency; BMI: body mass index; VO_2peak_: peak oxygen uptake; WC: waist circumference; PHV: Peak height velocity. * Statistically difference using the bootstrapping resampling procedure for the paired two-tailed *t-*test (p < 0.05)

Table 2 shows the longitudinal association between the interaction term of the CRF, measured by the estimation of VO_2peak_, and screen time with WC. A statistically significant interaction term was observed (B = -0.0003; 95% CI: -0.007; -0.0001), indicating that the relationship between screen time and WC varies across different CRF levels. Of note, a higher order interaction between CRF, screen time, and measurement period was tested, but the interaction term was not statistically significant (data not shown). This suggests that the association between screen time and CRF with WC remained consistent across the different time points of assessment.

**Table 2:** Longitudinal associations between the interaction of screen time and VO_2peak_ with WC.

	B	SE	*t*	*p*	95% CI
	WC (z-scores)				
Constant	0.096	0.089	1.074	0.283	-0.079; 0.270
Screen time	-0.0003	< 0.001	-2.625	0.009	-0.001; -0.001
VO_2peak_ (z-scores)	-0.988	0.056	-17.506	< 0.001	-1.099; -0.877
Interaction	-0.0003	< 0.001	-1.735	0.083	-0.007; -0.0001
PHV	-0.008	0.013	-0.592	0.554	-0.034; 0.018
Ethnicity	-0.016	0.060	-0.266	0.790	-0.134; 0.102
Measurement period	0.049	0.021	2.364	0.018	0.008; 0.089
	F (6, 795) = 159.201; *p* < 0.001; R^2^ = 0.546				

*Notes* – B: Unstandardized regression coefficients; SE: standard error; CI: Confidence interval; VO_2peak_: Peak oxygen uptake; PHV: Peak height velocity

**Fig. 1:**
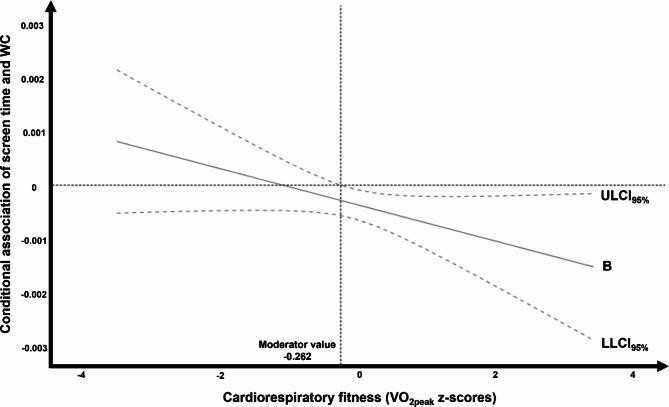
Conditional association of screen time and WC across different CRF values by the Johnson-Neyman technique. *Notes* – B: Unstandardized regression coefficient; ULCI: Upper limit confidence interval; LLCI: Lower limit confidence interval; VO_2peak_: Peak oxygen uptake

Additionally, the Johnson-Neyman technique was employed to estimate the regression coefficient of screen time and WC across different CRF levels (Fig. 1). It can be observed that when participants have a lower VO_2peak_, there is no association between screen time and WC. However, as the participants’ VO_2peak_ z-scores increase, the association between screen time and WC turns negative.

## Discussion

The main findings of the present study indicated that screen time and CRF interact in their relationship with WC and this interaction remains consistent at the two assessments. Briefly, the findings suggest that higher CRF can attenuate the harmful association that increased sedentary behavior might have on WC because when participants present a higher CRF, the relationship between screen time and WC is negative. These findings indicate the importance of motivating adolescents who engage in prolonged periods of sedentarism to adhere to physical activity and exercise recommendations [5], particularly those involving moderate-to-vigorous intensities. Doing so may effectively raise their CRF and energy expenditure, thereby reducing adiposity levels.

The cumulative impact of these unfavorable factors has an exponential effect on health. A survey involving schoolchildren demonstrated that those with low CRF levels exhibited a 15% increased risk of having elevated WC, irrespective of their screen time. When screen time was accounted for, these values increased to 24% [11]. Furthermore, an investigation with adolescents who had different CRF levels and screen time showed that those individuals with reduced CRF compared to the recommended and high screen time were three times more likely to develop a negative metabolic profile [24]. Another study, with schoolchildren from Southern Brazil, analyzed the joint association of CRF and screen time with the presence of metabolic risk and identified more prevalence of metabolic risk amongst those with low CRF levels, regardless of the presented screen time [25]. The presents findings agree with the aforementioned results because they suggest that achieving and maintaining healthier CRF levels may exert a protective role in the development of higher adiposity, even for those with higher amounts of sedentary behavior.

There is a clear need to promote the improvement of CRF among schoolchildren, as such behavior can serve as counterbalance to the excessive screen time. In our study, we observed that students improved their VO_2peak_ levels over time, but it may be a simple increase due to growth [26]. There are other determining factors for achieving and maintaining healthier CRF levels, such as the synergistic relationship with adiposity [14] and higher levels of fat-free mass [26]. The inclusion of physical education classes in school in addition to provision of suitable spaces and public policies to promote exercise among schoolchildren are essential for enhancing youth’s overall health by improving CRF. Given that screen time has become part of today’s society, it is essential to recognize that being physically active holds a positive association with CRF, independent of screen time [27].

The strengths of this study include the longitudinal follow-up of a randomly selected representative sample from a municipality in the Southern Region of Brazil, obtained at time-points over a period of 3-years. Regarding the limitations of the research, it would be pertinent to highlight the utilization of indirect for assessing sedentary behavior, cardiorespiratory fitness, and abdominal adiposity. Nevertheless, it is worth noting that indirect evaluations are accepted and employed by many epidemiological studies in the literature. Furthermore, the great loss of participants from baseline to follow-up is a limitation, as those who were not followed-up exhibited higher WC and screen time at baseline (data not shown; small standardized differences: *d* ≤ 0.28). The current study did not account for the time spent on other screen-based devices, such as mobile phones, which are frequently used by adolescents today. Therefore, caution should be exercised when comparing our findings to other studies that included other screen-based devices in their analysis. Lastly, there were no assessment of other confounding factors, such as physical activity and dietary uptake, and sedentary behavior, CRF, and WC can be influence by these variables.

## Conclusion

Screen time and CRF interact in their longitudinal association with WC from childhood to adolescence. Since screen time was contextualized as the independent variable, the results indicate that the relationship between screen time and WC varies across different CRF levels. These findings suggest that VO_2peak_ plays an important role in the relationship between screen time and WC. Therefore, for the purpose of achieving healthier abdominal adiposity levels, it is pertinent to considered the interactions between sedentary behavior and CRF. This finding highlights that, even though it is important to reduce screen time, the improvement of CRF is relevant to minimize the influence of screen time on WC.

## Data Availability

The database used and analyzed in the present study is not publicly available as its information may compromise the participants’ privacy and consent involved in the research. However, the data are available from the corresponding author, upon request.
